# Identification of newly developed advanced schistosomiasis with MALDI-TOF mass spectrometry and ClinProTools analysis

**DOI:** 10.1051/parasite/2019032

**Published:** 2019-06-05

**Authors:** Yuzheng Huang, Yongliang Xu, Yi Huang, Fang Sun, Haisong Tian, Nannan Hu, Liang Shi, Haiyong Hua

**Affiliations:** 1 National Health Commission Key Laboratory of Parasitic Disease Control and Prevention, Jiangsu Provincial Key Laboratory on Parasite and Vector Control Technology, Jiangsu Institute of Parasitic Diseases Wuxi 214064 Jiangsu Province PR China; 2 Public Health Research Center, Jiangnan University Wuxi 214122 Jiangsu Province PR China

**Keywords:** newly developed advanced schistosomiasis, MALDI-TOF mass spectrometry, ClinProTools, proteomic detection pattern

## Abstract

Cases of newly developed advanced schistosomiasis (NDAS) have occurred in areas where schistosomiasis transmission has been blocked for more than 25 years. The causes and pathogenesis of NDAS are still unknown. Diagnosis of NDAS relies on historical investigation and clinical symptoms, such as liver fibrosis, hepatic ascites and abnormal biochemical indexes in serum. It is important but difficult at this stage to develop a new tool for early screening and rapid diagnosis. In this study, serum peptides from thirty patients with NDAS and thirty healthy controls were captured with weak cation exchange magnetic beads, and subjected to MALDI-TOF mass spectrometry and ClinProTools analysis. Eleven peaks with m/z 924, 2661, 2953, 2991, 3241, 3884, 5337, 5905, 5943, 7766 and 9289 were decreased and three peaks with m/z 1945, 2082 and 4282 were increased in the NDAS group. The proteomic detection pattern (PDP) was established with 14 different peptide peaks, and its sensitivity and specificity were investigated with a blind test. The peptide mass fingerprints of sera from 50 NDAS patients and 100 healthy controls were double-blind subjected to the PDP method, and 50 patients and 92 healthy controls were classified as NDAS and healthy separately, which showed 100% sensitivity and 92% specificity. Our results showed that the PDP could be a new and useful method to detect NDAS.

## Abstract (fr)

Des cas de schistosomiase avancée récemment développée (SARD) se sont produits dans des zones où la transmission de la schistosomiase est bloquée depuis plus de 25 ans. Les causes et la pathogenèse de la SARD sont encore inconnues. Le diagnostic de la SARD repose sur des recherches historiques et des symptômes cliniques, tels que la fibrose hépatique, l’ascite hépatique et des indices biochimiques anormaux dans le sérum. Il est important, mais difficile à ce stade, de développer un nouvel outil de dépistage précoce et de diagnostic rapide. Dans cette étude, les peptides sériques de 50 patients atteints de SARD et de 30 témoins sains ont été capturés avec des billes magnétiques à échange de cations faibles, soumis à une spectrométrie de masse MALDI-TOF et à une analyse par ClinProTools. Onze pics avec m/z 924, 2661, 2953, 2991, 3241, 3884, 5337, 5905, 5943, 7766 et 9289 étaient diminués et trois pics avec m/z 1945, 2082 et 4282 étaient augmentés dans le groupe SARD. Le modèle de détection protéomique (PDP) a été établi avec 14 pics de peptide différents, et sa sensibilité et sa spécificité ont été étudiées avec un test à l’aveugle. Les empreintes peptidiques des sérums de 50 patients atteints de SARD et de 100 témoins sains ont été soumises à la méthode du PDP à double insu. Cinquante patients atteints et 92 témoins sains ont été classés séparément en tant que SARD et sains, ce qui démontre une sensibilité de 100 % et une spécificité de 92 %. Nos résultats ont montré que le PDP pourrait être une méthode nouvelle et utile pour détecter la SARD.

## Introduction

Schistosomiasis is one of the most important and severe parasitic diseases in tropical and subtropical areas [7, 19]. Worldwide, it affects >200 million people, with 779 million at risk of infection, and causes the loss of an estimated 4.5 million disability-adjusted life years [20, 28]. The final outcome of schistosomiasis is advanced schistosomiasis [6], which can lead to a serious life-threatening condition. Control of transmission of endemic schistosomiasis has been achieved in many areas in China, especially in Jiangsu province from 2011 [2]. Simultaneous treatment and relief of advanced schistosomiasis is well documented [25]. Some cases have occurred in areas where schistosomiasis transmission has been blocked for >25 years [3, 24]. These new cases were named newly developed advanced schistosomiasis (NDAS) by an expert group meeting on schistosomiasis in 2013 and the pathogenesis of NDAS needs further study [5]. Previously, we investigated biochemical indexes and serum proteomic profiles of NDAS, which showed liver fibrosis and some differences in serum [26].

Mass spectrometry can be used to search for biomarkers in the body fluids [8, 9] of patients with diseases such as Chagas disease (*Trypanosoma cruzi*) [16] and *Entamoeba histolytica* infection [15], as well as in rheumatoid arthritis screening [12] and cancer research [1, 9, 12, 27]. Mass spectrometry has many advantages, including high sensitivity, short duration from sample collection to diagnosis, potential for high-throughput screening, and a requirement for only small biological samples [10]. We have previously used ClinProTools with matrix-assisted laser desorption ionization time-of-flight mass spectrometry (MALDI-TOF-MS) and magnetic-beads-based weak cation exchange chromatography for enrichment and purification of peptides and proteins [4, 6]. Serum peptides differ as early as nine days post-infection in rabbits with *Schistosoma japonicum* infection, and ClinProTools has also been used for early detection of schistosomiasis in sentinel mice.

**Figure 1:**
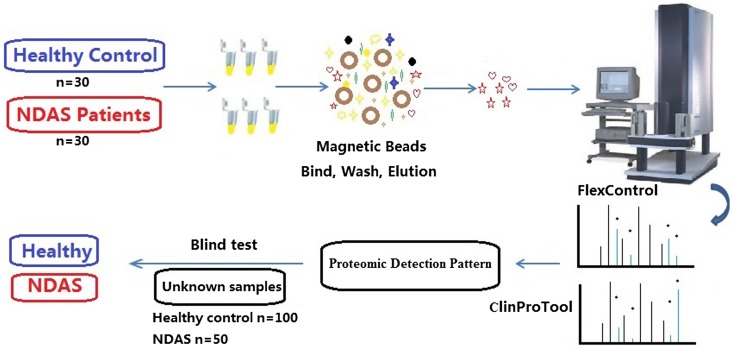
The study design, including sample collection and preparation, MALDI-TOF MS and blind test.

In the present study, we designed a laboratory assay to measure differentially expressed peptides in serum from NDAS and healthy controls using MALDI-TOF MS and ClinProTools, as shown in Figure 1. The selected peaks were applied to generate proteomic detection pattern (PDP) for NDAS identification.

## Materials and methods

### Patient identification and healthy controls

We enrolled patients infected with *S. japonicum* from the Jiangsu provincial census record for detection and treatment, which was compiled in 1977 and checked four times in 1987, 1997, 2008 and 2012. Patients were diagnosed with NDAS according to the sera biochemical index, clinical symptoms and medical history as follows: the serological criterion with hyaluronic acid (HA) > 100 ng/mL and γ-glutamyl transferase (GGT) > 50 U/L; network-like changes in the liver and portal vein widening with B-ultrasound scan (Table 1); and historical investigation in patients infected with *S. japonicum* before the 1970s and treated with antimony or hexa-chloro-p-xylol four times, with pyquiton at the last chemotherapy. All patients underwent fecal examination 11–45 times from 1976 to 1985 and all results were negative. According to the patients’ self-report, their symptoms including pain in the liver appeared and gradually increased from 2008. Thirty NDAS patients aged 55–75 years were selected with the diagnostic criteria, and the healthy control group of thirty age-matched individuals was set as a negative control. A group of an additional fifty NDAS patients and one hundred health controls, following the reporting guideline STARD (Sup. 1 and Sup. 2), were collected for PDP evaluation. All the healthy controls and NDAS patients were evaluated by physical examination at hospital; other diseases including fatty liver, alcohol-induced liver injury, and hepatitis B and C virus infection were excluded.

**Table 1:** Newly developed advanced schistosomiasis (NDAS) patients identified by historical survey, liver B-ultrasound scan and serological examination, 30 healthy people were selected as negative controls.

Case	Number	Age	HA (ng/mL)	GGT (U/L)	B-ultrasound	Time interval between the last chemotherapy and advanced Schistosomiasis determined (years)
NDAS	30	68.49 ± 7.10	484.97 ± 458.07	168.07 ± 130.35	Network-like changes in liver and portal vein widening	35.6 ± 5.04
Healthy control	30	68.79 ± 4.10	50.67 ± 16.46	32.53 ± 7.69	No obvious abnormality in the liver	–

**Table 2:** Fourteen differential peaks were detected in sera from newly developed advanced schistosomiasis (NDAS) cases when compared to sera from healthy controls using MALDI-TOF-MS and ClinProTools analysis.

m/z	Peak amplitude		*p*
	Control group	NDAS group	
5905	775.00 ± 425.26	265.1 ± 205.1	0.00317
5943	70.98 ± 44.01	22.14 ± 6.53	0.00317
2953	62.49 ± 31.99	28.06 ± 10.8	0.00317
2082	51.67 ± 37.16	173.00 ± 120.97	0.0054
3884	51.28 ± 29.95	28.85 ± 13.22	0.024
1945	93.94 ± 73.26	205.9 ± 142.84	0.0249
4282	52.79 ± 90.67	235.4 ± 245.63	0.0254
924	98.45 ± 85.43	40.57 ± 17.4	0.0276
5337	116.8 ± 83.75	60.43 ± 46.13	0.0423
3241	108.7 ± 52.58	76.59 ± 27.40	0.0496
9289	165.82 ± 124.86	90.63 ± 63.89	0.0495
7766	297.88 ± 192.94	178.61 ± 121.2	0.0494
2661	119.30 ± 129.47	69.27 ± 37.72	0.0493
2991	76.9 ± 79.66	42.4 ± 55.15	0.0490

*Note*. Data are expressed as mean ± *SD*.

### Sample collection, HA and GGT detection

Blood was collected from the arm vein of thirty NDAS patients, left to clot for 30 min at room temperature, and centrifuged at 3000 ×*g* for 10 min at 4 °C to obtain the serum. Sera from thirty healthy controls were used as negative controls. The serological concentration of HA was measured with sandwich ELISA (Elabscience Biotechnology Co. Ltd., Wuhan, China). The standard and unknown samples were added to the plate wells, where the HA-specific antibody was pre-coated. After incubation and washing, biotinylated detection antibody specific for HA and avidin–horseradish peroxidase-conjugate was added to each well. The optical density (OD) was measured immediately at 450 nm after the stop solution was added. The standard curve was drawn with the concentration of standard samples and OD values and the concentrations of HA in the unknown samples were calculated according to the standard curve. GGT was detected with an automatic biochemical analyzer (COBAS C501; Roche). Serum samples from fifty NDAS patients and one hundred healthy controls were collected from a local community health service center in Changshu County, Jiangsu Province, China.

### MALDI-TOF MS

The study design is shown in Figure 1. The MB-WCX Profiling Kit (Bruker Daltonics, Bremen, Germany) was used for serum peptide enrichment, as described previously [4]. Magnetic beads and serum were mixed in a binding solution, followed by 2 min incubation at room temperature in a standard thin-walled polymerase chain reaction tube. After thorough mixing, washing and elution, the treated sample was transferred to a fresh tube containing 5 μL stabilization buffer. After combination with the matrix of α-cyano-4-hydroxycinnamic acid (Sigma, St. Louis, MO, USA), the mixture was spotted on the polished target (Bruker Daltonics) and detected with MALDI-TOF MS (Autoflex III; Bruker Daltonics). Four MALDI spots of each sample were measured with FlexControl software (Bruker Daltonics). For each spot, 1000 spectra were acquired (200 laser shots at five different spot positions) and all spectra with a signal-to-noise ratio > 3 were recorded for peptide mass fingerprinting. Mass accuracy was calibrated before measurements, and the detection parameters were set according to the manufacturer’s instructions.

### Data processing and analysis

ClinProTools was used to facilitate the processing and comparison of multiple spectra by automatically defining peak values, normalizing data, subtracting baseline values, and recalibrating the data. Our previous study gave more details [4]. The PDP was established with the differential peaks amplitude to distinguish NDAS and healthy controls with ClinProTools software, as described previously [4, 6]. The peak amplitude was shown as mean ± *SD*, and statistical analysis relied on Welch’s *t*-test. A *p* value < 0.05 was considered significant and *p* < 0.01 highly significant.

### Blind test with PDP

Once the proteomic pattern had been established, the output value for healthy controls was set as 0, that for patients as 1. The additional samples of healthy control and NDAS collected were scored “0” or “1” by the technician without prior knowledge of their true status. The serum samples of fifty NDAS patients and one hundred healthy humans who lived in the same villages were collected and treated with the MB-WCX Profiling Kit, as described above. All spectra captured by MALDI-TOF MS were imported into ClinProTools software for spectrum classification and post-processing, and PDP was used for sample identification. Fifty NDAS patients for the blind test were compared with the thirty NDAS patients for PDP, showing no significant difference (*p* > 0.05) (Table 3).

**Table 3:** Comparison of newly developed advanced schistosomiasis (NDAS) patients between the training group and the verification group.

Characteristics	No. of cases		*p*
	NADS for PDP	NADS for blind-test	
Total cases	30	50	
Gender			0.525
Male	16	23	
Female	14	27	
Age			0.262
HA (0–100 ng/mL)	484.97 ± 458.07	541.52 ± 482.56	0.576
GGT (3–50 U/L)	168.07 ± 130.35	142.98 ± 109.19	0.358

### Sensitivity, specificity and reproducibility assay

Sensitivity and specificity were assessed for all the spectra of fifty NDAS patients and one hundred healthy humans with the established PDP. Species specificity was also investigated with twenty sera from *Toxoplasma gondii* infections. The reproducibility and efficiency of MB-WCX bead fractionation were estimated by measuring all samples three times. Each sample was an aliquot stored at −80 °C and was used only once.

## Results

### Serological detection

HA and GGT were measured in all the patients’ sera. The concentrations of HA and GGT were 115.19–1327.8 ng/mL and 53–586 U/L, respectively, and 29–80 ng/mL and 22–48 U/L in healthy controls; the data for the groups is shown in Table 1. The reference values of HA and GTT in the sera of healthy controls were 0–100 ng/mL and 0–50 U/L, respectively.

### PDP

**Figure 2:**
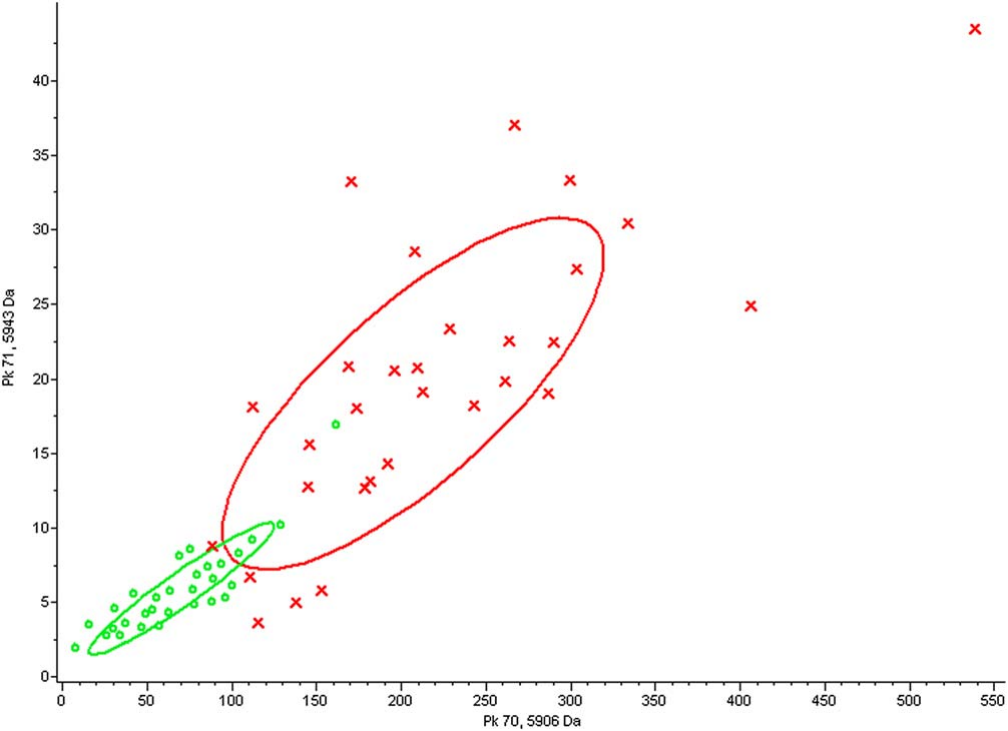
Two-dimensional peak distribution with the peptide mass fingerprint from the newly developed advanced schistosomiasis (NDAS) and control groups. The red cross indicates healthy controls and the green circle indicates newly developed advanced schistosomiasis (NDAS) patients.

**Figure 3:**
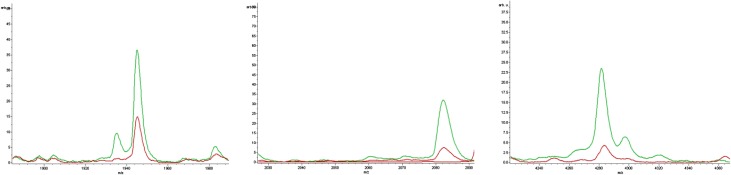
Peptide peaks with features of up-regulation. Compared with the healthy controls (red line), the increased peaks of the newly developed advanced schistosomiasis (NDAS) group are shown as a green line, including three peaks with m/z 1945, 2082 and 4282.

**Figure 4:**
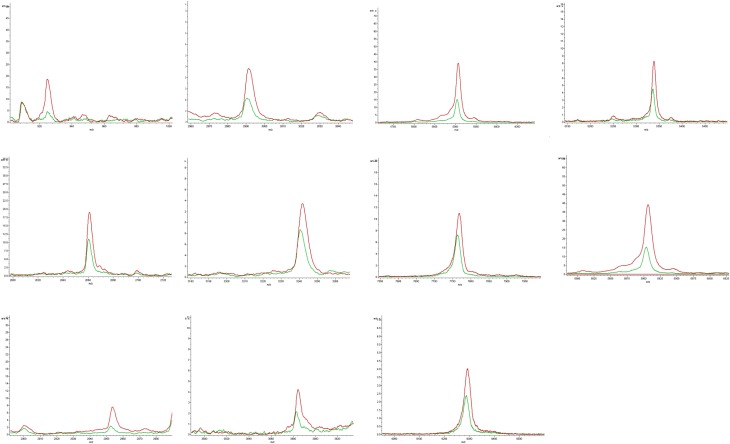
Peptide peaks with features of down-regulation. Compared with the healthy controls (red line), the down-regulated peaks of the newly developed advanced schistosomiasis (NDAS) group are shown as a green line, including 11 peaks with m/z 924, 2661, 2953, 2991, 3241, 3884, 5337, 5905, 5943, 7766 and 9289.

Sera from NDAS patients and healthy controls were collected and treated with an MB-WCX Profiling Kit, followed by detection with MALDI-TOF MS and analysis by FlexControl and FlexAnalysis software. The two groups were separated into two clusters with two-dimensional peak distribution analysis, as shown in Figure 2. ClinProTool analysis revealed that 11 peaks with m/z 924, 2661, 2953, 2991, 3241, 3884, 5337, 5905, 5943, 7766 and 9289 were decreased and three peaks with m/z 1945, 2082 and 4282 were increased in the NDAS group, with a significant difference (*p* < 0.05, *p* < 0.01) in the amplitudes and peak resolution ratios, as shown in Table 2, and which were chosen for PDP establishment. The increased and decreased peaks are shown in Figures 3 and 4 with the average altitude.

### Sensitivity, specificity and reproducibility of PDP

The spectra from the other 50 NDAS patients were subjected to PDP and all were successfully verified, which showed 100% sensitivity. One hundred healthy humans who lived in the same villages were also blind tested with PDP for specificity investigation, and 92 were verified correctly. The sera from the patients infected with *T. gondii* were also checked; all sera from patients with *T. gondii* infection were classified “0” (= not infected) using the criteria for NDAS detection established by MS and none of the cases could be diagnosed as NDAS, which indicated 100% species specificity. For the evaluation of reproducibility and efficiency, MB-WCX fractionation was assessed by measuring all samples in triplicate, and all the spectra showed the uniformity for the same sample.

## Discussion

Hyaluronic acid (HA) is a high molecular weight glycosaminoglycan produced by fibroblasts throughout the body and plays a structural role in the connective tissue matrix. HA is recognized as a non-invasive marker and gold standard of liver fibrosis in chronic liver diseases [17]. According to the census records, all patients infected with *S. japonicum* in Kunshan County received chemotherapy in 1976 [3]. General investigation of the whole population in 1977 and 1983 and physical re-examination in 1979, 1987, 1992 and 1997 revealed that several patients were cured of schistosomiasis, and others were diagnosed with advanced schistosomiasis. The latter had many clinical symptoms such as liver pain, network-like changes in the liver, portal vein widening upon B-ultrasound scanning, and liver fibrosis, which sometimes required surgical treatment. However, some of the patients with apparently cured schistosomiasis reported aggravation of symptoms and they met the criteria for advance schistosomiasis. This indicates that the disease has a longer time of progression and a complicated mechanism of occurrence, and development of the disease is unclear. This new type of schistosomiasis was named NDAS by an expert group [5].

A report on endemic schistosomiasis revealed that the last *Oncomelania hupensis* snail was found in 1983 and eradication of schistosomiasis was achieved in 1989 in Kunshan County [26]. There was no opportunity for re-infection of humans and livestock [7]. Investigation of life history showed that NDAS patients lived in the local area from the 1980s and had no re-exposure to water contaminated with Schistosoma larvae. Hence, NDAS seems not to be related to *S. japonicum* and may be attributed to a different pathogenesis.

Some studies have shown that praziquantel, the primary drug in schistosomiasis treatment [13, 18], can only kill adult worms and has no effect on the eggs of *S. japonicum*, which results in pathological damage from the eggs that persist in the liver after insecticidal treatment [11, 14]. The soluble egg antigen that is released by the eggs induces liver granuloma and fibrosis, which are the main symptoms in advanced schistosomiasis [22]. However, the eggs could only survive in the mammalian body for about 21 days. The question is how the pathological process can last for the next 10–30 years? The pathogenesis of liver fibrosis without living parasite eggs is still unclear, as is the function of dead eggs in the process.

China has set 2020 as a date for elimination of schistosomiasis [21]. ELISA for antibody detection in serum and microscopy in fecal examination are the main methods to detect *S. japonicum* infection [23]. However, ELISA showed positive results even in treated patients with no eggs found by fecal examination for many years, which led to difficulty in the identification of patients. Fecal examination is a gold standard method for schistosomiasis detection, which can be confirmed once eggs are found in the feces by microscopic detection However, the method has low sensitivity and may lead to false-negative diagnosis, especially with the low infectivity during schistosomiasis elimination.

The PDP established in this study represents a new method to distinguish between NDAS and healthy controls. The results showed that the method worked well with our selected samples, and separate clusters indicated that there were different peak distributions in the two groups. In this study, the fourteen peaks with m/z 924, 2661, 2953, 2991, 3241, 3884, 5337, 5905, 5943, 7766, 9289, 1945, 2082 and 4282 were found in sera with MALDI-TOF MS technology, and were different from the peaks in sera of infected mice from our previous study [6]. Part of the reason could be attributed to the different species and host-parasite interactions. The different peaks in this study could hopefully be used as diagnostic biomarkers, especially m/z 5905, 5943, 2953 and 2082 with *p* < 0.01 by *t*-tests. Although the unknown origin of the peaks was a technical limitation in our study, the PDP established with these peaks was useful to distinguish the selected samples. The blind test showed 100% sensitivity (50/50), 92% (92/100) specificity and accuracy in detection of the unknown samples, and 100% species specificity using sera associated with *T. gondii* infection. However, it is suggested that the PDP could not be directly used for classification of NDAS and healthy controls on a larger scale at this stage and more samples are needed for further verification. The limitations of our study were the absence of specific biomarkers, and the small sample size, and we should collect more cases in future research. In patients with NDAS, regardless of the pathological and etiological mechanism, the emergence of NDAS also suggested that treatment of liver fibrosis cannot be ignored after chemotherapy.

## Ethics statement

All experiments described here fully complied with current national and institutional regulations and with those of the Ministry of Science and Technology of the People’s Republic of China.

## Author contributions

YH and HH conceived and designed the experiments; YH, NH and YX performed the experiments; YH, HH, NH and LS analyzed the data; YH, FS, HT and YH collected the samples; all authors read and approved the final manuscript.

## Funding

This study was supported by the Natural Science Foundation of China (Grant Nos. 81673673, 31201893), the Special Program of Jiangsu Clinical Medicine (Grant No. BL2014020), Jiangsu Province’s Key Medical Center (Grant No. 201108), the Natural Science Foundation of Jiangsu Province (Grant No. BK2011164), the Jiangsu Health Science Project (Grant Nos. X201416, X201110 and X201505), a project of the Public Health Research Center of Jiangnan University (Grant Nos. JUPH201811 and JUPH201802), Jiangsu Health International Exchange Program to Y Huang, and by the Project of Invigorating Health Care through Science, Technology and Education. The funders had no role in study design, data collection, management, analysis and interpretation, decision to publish, or in the preparation, review or approval of this manuscript.

## Competing interests

The authors declare that they have no competing interests.

## Supplementary materials

Supplementary material is available at https://www.parasite-journal.org/10.1051/parasite/2019032/olm. Supplement 1 Flow diagram of the patient population and reasons for exclusion. NDAS, newly developed advanced schistosomiasis. Supplement 2 Flow diagram of the healthy control population and reasons for exclusion.
