# Supplementary figures and images for: Identification of newly developed advanced schistosomiasis with MALDI-TOF mass spectrometry and ClinProTools analysis
Source: Parasite. 2019 Jun 5;26:33. doi: 10.1051/parasite/2019032 (PMC6550559; doi:10.1051/parasite/2019032)

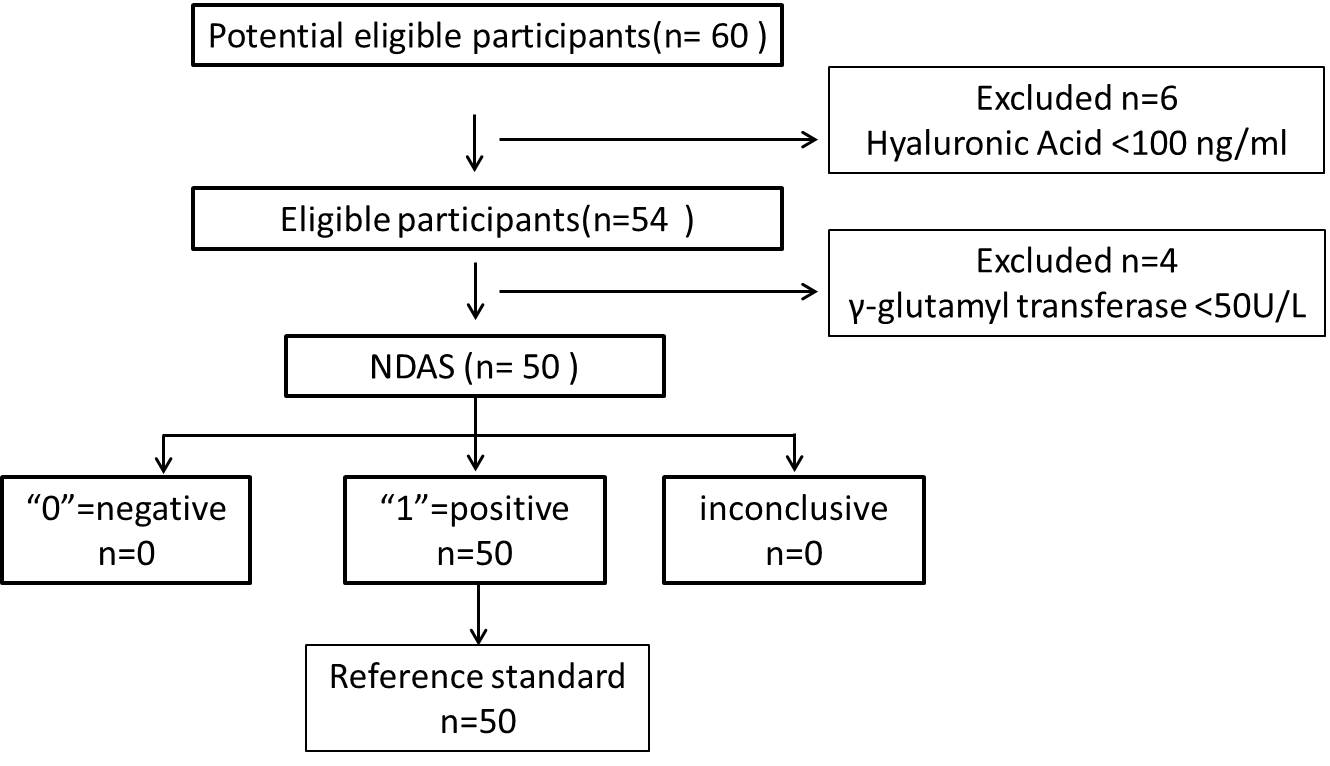

Supplement: Supplement 1 — Flow diagram of the patient population and reasons for exclusion. NDAS, newly developed advanced schistosomiasis. [file parasite-26-33-s1.jpg]

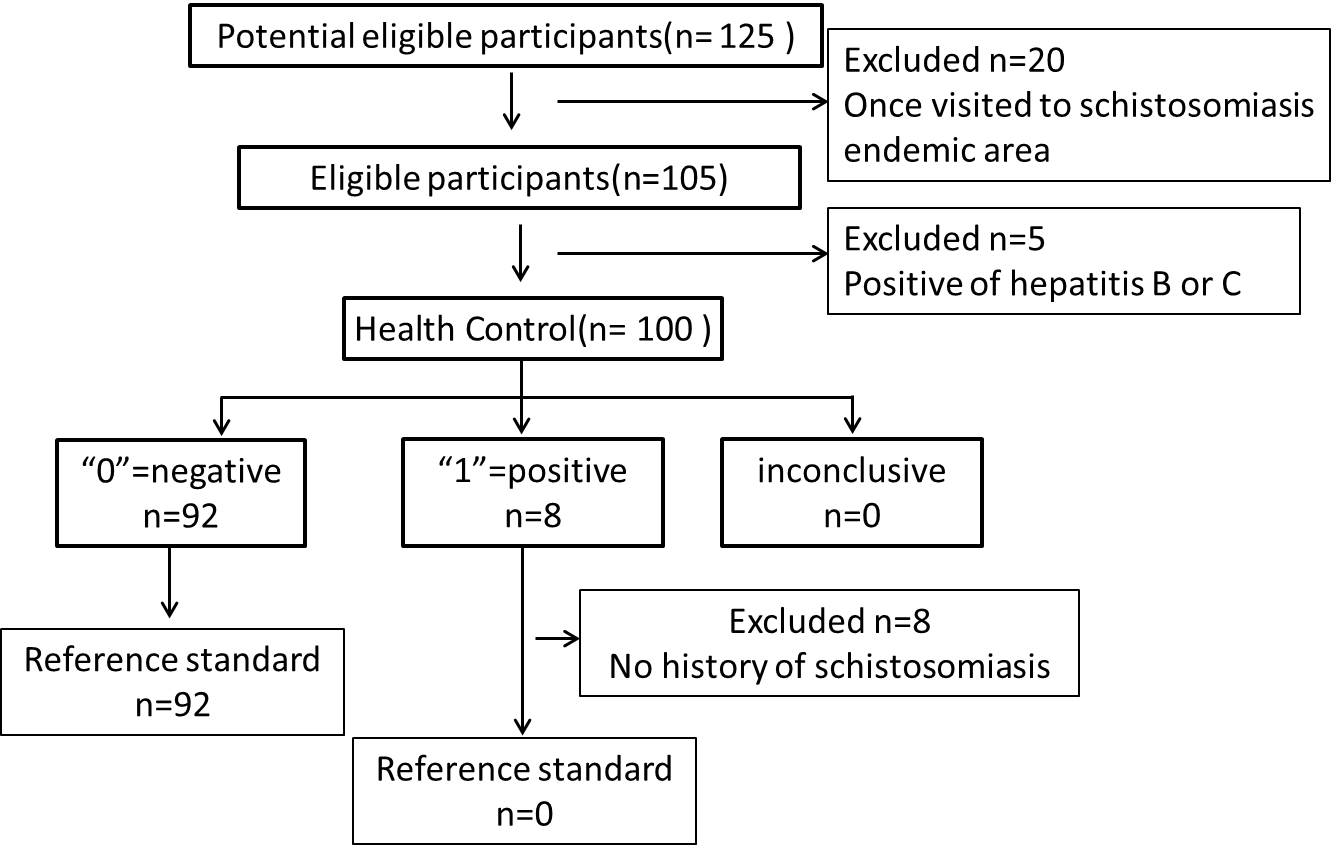

Supplement: Supplement 2 — Flow diagram of the healthy control population and reasons for exclusion. [file parasite-26-33-s2.jpg]
